# Intervention Effects of a Kindergarten-Based Health Promotion Programme on Motor Abilities in Early Childhood

**DOI:** 10.3389/fpubh.2020.00219

**Published:** 2020-06-30

**Authors:** Susanne Kobel, Lea Henle, Christine Laemmle, Olivia Wartha, Bertram Szagun, Juergen Michael Steinacker

**Affiliations:** ^1^Division of Sports and Rehabilitation Medicine, Department of Internal Medicine, University Hospital Ulm, Ulm, Germany; ^2^Faculty Social Work, Health & Nursing, University of Applied Sciences Ravensburg-Weingarten, Weingarten, Germany

**Keywords:** physical activity, children, preschool, endurance performance, socio-economic status

## Abstract

**Background:** Physical activity is positively related to motor abilities. Especially in childhood, an active lifestyle is important to support healthy motor development. The low-threshold health promotion programme “Join the Healthy Boat” in kindergartens promotes physical activity in order to also improve motor abilities. Here, effects of the programme on children's motor abilities after 1 year were investigated.

**Materials and Methods:** The longitudinal study included 419 children (3.7 ± 0.6 years) from 58 kindergartens throughout south-west Germany (intervention: 254, control: 165). Children in the intervention group received physical activity promotion with a focus on motor ability development, led by teachers, through one kindergarten year; children in the control group followed the normal kindergarten routine. At baseline and follow-up, motor tests (3-min-run, one-leg-stand, standing long jump, sit-and-reach-test) were performed, anthropometric measures (body weight and height) were taken and a parental questionnaire was issued. Intervention effects were assessed using differential measures (follow-up – baseline) adjusted for gender, age, socioeconomic status (SES) and baseline values, with covariance analyses.

**Results:** Children in the intervention group showed a significant improvement in endurance performance $\left(F\left(1.329\right)=20.95,\;p<0.000,\;\eta_{P}^{2}=0.060\right)$, which applies to boys $\left(F\left(1,172\right)=13,66,\;p\leq 0.000,\;\eta_{P}^{2}=0,074\right)\;$ and girls $\left(F\left(1,152\right)=7,48,\;p\leq 0.007,\eta_{P}^{2}=0,047\right)$. No significant intervention effects on endurance performance were found for children with low baseline values, children with a low SES, and children aged 5 years, nor for any other assessed motor ability.

**Conclusions:** The theory-based, teacher-centered intervention promoting physical activity in order to also improve motor abilities has shown a positive effect on endurance performance in kindergarten children, but no other motor ability. Future interventions should therefore be either longer, more intense and take into account children's age, initial level of performance and their SES. In addition, the influence of teachers should be considered more closely in future research.

## Introduction

From an early age, physical activity supports a healthy growing-up as well-children's development of their motor abilities (1, 2). However, research shows that physical activity levels of children have declined in recent years, and that only half of kindergarten-aged children are sufficiently physically active (3–5). In Germany, 43% of 3–6-year-old girls and 49% of the boys are moderately to vigorously physically active for at least 1 h per day (4). These global recommendations [moderate to vigorous physical activity for at least 1 h per day (6)] can only be seen as a minimum of daily physical activity (6). Children between the ages of three and four (6) and 4 and 6 years of age (7) are encouraged to engage in a minimum of 180 min of physical activity per day, respectively, which includes structured and unstructured activities (7). In general, it is advised to reduce periods of inactivity and increased levels of physical activity have shown to result in greater health benefits such as prevention of obesity, developing an active lifestyle throughout childhood, adolescence and into adulthood, as well as greater cognitive and academic achievements (4, 8).

It is undisputed that physical activity and motor abilities are interrelated (9). Developing adequate motor abilities is considered as one of the key developmental tasks in early childhood (10), for health-related fitness, the promotion of physical activity as well as the prevention of overweight and sedentary behavior (11–13). In addition, studies were able to show that the level of motor abilities during childhood is positively associated with later physical activity behavior (14). Furthermore, a positive correlation between motor abilities and various developmental areas, such as cognitive abilities and language acquisition have been suggested (15–17).

Along with the decline in physical activity in recent decades, various studies also indicate a change in motor abilities in childhood and adolescence (12, 18, 19). For children at kindergarten age, there are only very few evidence-based studies that have addressed changes in motor abilities over recent years. Overall, the results available to date do not show a uniform picture; compared to previous generations, significant changes can only be seen in individual motor abilities, especially in the energetic-conditional area and flexibility (10, 20, 21).

The positive impact of physical activity on children's motor abilities, health and health behaviors is subject of ongoing health research. Findings of various evaluation studies of physical activity promotion measures suggest that targeted promotion of physical activity can positively support the development of motor abilities even at kindergarten age (22–27). Therefore, it is recommended to integrate physical activity promotion into children's everyday lives from an early age on in order to promote a physically active lifestyle and encourage more movement experiences so motor abilities can be developed (4, 28–31).

Since it is one of the first educational institutions children enter, kindergarten is ideally suited for early support of health resources, as it is possible to realize behavior as well as environmental change. In Germany, visiting a kindergarten is voluntary, still, almost all children – independent of their social background – between the ages of three and six can be reached here (32, 33). It is primarily used as a child care offer, not comparable with school, i.e., without fixed curriculum but some recommendations of promoting certain developmental areas during the time children are at kindergarten (e.g., knowledge, creativity, motor skills etc.). The theory-guided health promotion programme “Join in the Healthy Boat” aims to intervene in that setting and offers kindergarten teachers materials to realize bespoke recommendations in health-related areas. The intention of this low-threshold behavioral and preventive intervention is to promote children's health behaviors in kindergartens (34) with the focus on physical activity, nutrition and leisure time activities in order to inter alia improve children's motor abilities. Without adding extra lessons or interfering too much, the teacher-based programme supports and structures already present elements of the daily kindergarten routine such as educational lessons, physical activity sessions, and trips into more health promoting ones. Against the background of the already identified need for early promotion of physical activity and motor abilities, this study investigated intervention effects of the health promotion programme on kindergarten children's motor abilities.

## Materials and Methods

### Study Design

For this cluster randomized longitudinal study, nearly 8,000 kindergartens in south-west Germany were contacted by mail. 398 kindergarten teachers of 66 kindergartens provided written, informed consent for participation in the study. After that, a three-stage randomization on the basis of kindergarten size [see (34)] was performed to assign kindergarten children to intervention or control group, which resulted in a drop-out of 22 kindergarten teachers and therefore eight kindergartens. More detailed information can be found elsewhere (34).

The framework-guided (35) study was conducted in kindergartens throughout south-west Germany. Before and after the intervention period of 1 year, age-appropriate tests and measurements were carried out in the kindergartens of all participating children whose parents had given their written informed consent and children their assent. After those examinations, a parental questionnaire was issued. After baseline measurements were completed, the kindergartens were divided into intervention and control group. Kindergartens in the intervention group implemented the “Healthy Boat” programme during one kindergarten year; the control group carried on with their normal kindergarten routine (starting with the programme after follow-up measurements were completed). However, it has to be assumed that children in the control group also received some kind of health-promotion since the Ministry of Culture, Youth and Sport recommends health promotion at kindergartens, however does not give any guidance on how to implement it (32).

The theory-based intervention (36, 37) consists of weekly exercises and games lessons with the focus to improve children's motor abilities, also includes ready-to-use ideas, action alternatives, and instructions to get children to be more physically active and gain knowledge about their body and health, as well as eat more healthily (38). Additionally, ideas on how to re-arrange rooms and outdoor spaces for more opportunities to be physically active as well as helpful structures and norms were presented to enable the development of healthy eating habits. Further, short activity games (5–7 min each) designed to promote children's physical activity and motor abilities were incorporated into the daily kindergarten routine and delivered by the kindergarten teachers (more details: 34).

The here reported results were assessed as a secondary outcome of the programme. Primary outcomes as well as other secondary aspects of the programme are reported elsewhere (39–42).

### Instruments

Children's flexibility (sit-and-reach), balance (one-legged stand), and speed strength (standing long jump) were assessed using the KiMo-Test (43), children's endurance performance was assessed during a 3-min run (44). Skilled examiners carried out the tests on a one-to-one basis, including trained students who recorded the results.

During a kindergarten visit, children's height (m) and body weight (kg) were measured to ISAK standards (45) by trained staffed using a stadiometer and calibrated electronic scales (Seca 213 and Seca 826, respectively, Seca Weighing and Measuring Systems, Hamburg, Germany). Based on height and weight, children's body mass index (BMI; kg/m^2^) was calculated and subsequently converted to BMI percentiles based on German reference data (46). Children thereafter were classified into under-/normal weight (percentiles <90), overweight (percentiles ≥ 90) and obese (percentiles ≥ 97).

Socio-demographic information as well as children's and parental health behaviors were collected via parental questionnaire. For the present study, data on children's physical activity behavior {“On how many days of a normal week is your child physically active for a total of at least 60 min a day?” [WHO guideline (6)]} was derived from bespoke questionnaire, as well as their leisure time activities (“Is your child physically active in/out of a sports club?”). In order to calculate children's socioeconomic status (SES), based on the so-called Winkler index (47), highest parental education level, their occupation and the household income were used. This was followed by a division into a low, medium and high SES.

### Statistical Analyses

For the performed analyses, a significance level of α ≤ 5% was defined; data were calculated using SPSS Statistics 25 (SPSS Inc., Chicago, IL, US). Effect strength was evaluated using the partial eta square $(\eta_{P}^{2})\;\geq 0.059$. To check randomization, the two groups were screened for possible differences based on various characteristics. For nominally scaled variables chi-square tests; for ordinal scaled variables, Mann-Whitney *U*-tests, and for metric variables, *t*-tests for independent samples were used. In addition, *t-*tests for dependent samples were used to analyse within group effects. The differential measures (follow-up - baseline) of all dependent variables (motor tests) were checked for existing differences and analyzed by covariance analyses (ANCOVA). Based on the current state of research, the factors intervention/control group, gender, age, and SES were included in the statistical model in order to examine intervention effects; participation in organized sports was originally planned to be included as well but showed no effect in any models, so it was disregarded. In the case of intervention effects, the results were stratified for different characteristics.

## Results

### Sample Descriptives

After plausibility checks, the valid sample included 558 children from 58 kindergartens. Since only children who had completed the full motor test battery at baseline and follow-up were included, 139 children were excluded from the dataset and the thereafter analyzed sample consists of 419 children (254 in the intervention group, 165 in the control group) from 53 kindergartens (ranging from 3 to 30 children per kindergarten).

Table 1 shows the sample's baseline characteristics; the two groups differ significantly in their composition in terms of gender (χ^2^ = 9.95; *p* = 0.002). For their motor abilities, at baseline, the children in both groups achieved similar levels of endurance performance (3-min run) as well as flexibility (sit-and-reach) and lower limb strength (long jump). For balance (one-legged stand) however, there was a significant difference at baseline with children in the intervention group performing significantly better than those in the control group [*t*_(417)_ = 3.19, *p* = 0.002].

**Table 1:** Baseline characteristics of the total sample, intervention and control group.

	**Missing values**	**Intervention group**	**Control group**	**Total sample**
Number (n; %)		254 (60.6)	165 (39.4)	419 (100)
Gender [boys] (n; %)^a^		140 (55.1)	74 (44.8)	214 (51.1)
Age [years] (m; sd)		3.7 (0.6)	3.7 (0.5)	3.7 (0.6)
Body weight [kg] (m; sd)	7	17.4 (2.4)	17.4 (2.8)	17.4 (2.6)
Height [cm] (m; sd)	6	105.6 (5.8)	105.8 (5.4)	105.0 (5.6)
BMI percentiles (m; sd)	7	51.1 (25.6)	47.9 (26.2)	49.8 (25.9)
Overweight/obese (n; %)	7	14 (5.6)	9 (5.5)	23 (5.6)
Socio-economic status (n; %)	83			
Low		19 (9.4)	13 (9.8)	32 (9.5)
Medium		67 (33.0)	46 (34.6)	113 (33.6)
High		117 (57.6)	74 (55.6)	191 (56.8)
**Physical activity**				
Physically active during leisure time (organized and unorganized) (n; %)	67	175 (82.5)	105 (75.0)	280 (79.5)
Participation in organized sports (n; %)	19	121 (51.1)	83 (50.9)	204 (51.0)
MVPA for at least 1 h/day [n days] (m; sd)	83	2.7 (2.0)	2.3 (1.8)	2.5 (2.0)
MVPA for at least 1 h/day on most (i.e., 4 or more) days/week (n; %)	83	57 (27.5)	27 (20.9)	84 (25.0)
**Motor abilities at baseline (m; sd)**				
3-min run [m]		253.8 (48.6)	257.8 (43.9)	255,3 (46.8)
One-legged stand [n floor contacts]^b^		18.4 (8.6)	21.2 (9.0)	19.5 (8.8)
Sit-and-reach [cm]		2.7 (4.7)	2.3 (6.0)	2.5 (5.2)
Standing long jump [cm]		67.6 (20.0)	63.6 (21.2)	66.0 (20.5)

a significant difference between both groups (p = 0.04);

b *significant difference between both groups (p = 0.002)*.

*n, number; m, mean; sd, standard deviation; overweight/obese, BMI percentiles ≥ 90; MVPA, moderate to vigorous physical activity*.

### Association of Physical Activity and Organized Sports

At baseline, there was a significant difference for meters run during 3 min between those children who were physically active for a minimum of 60 min per day on at least 4 days per week and those who are not [*t*_(334)_ = −2.29, *p* = 0.02]. Children who are physically active on most days per week for 1 h achieved 13.5 m more than those who are less physically active (266.7 (± 47.7) m vs. 253.3 (± 46.3) m, respectively). This effect however, could not be observed for any other motor ability, nor for children's organized sports participation on any of the tested motor variables or at follow-up.

### Intervention Effects

After 1 year, both groups improved their endurance performance significantly as well as their balance and lower limb strength. A decrease in trunk flexibility was evident in both groups, however only significant in the intervention group.

**Figure 1:**
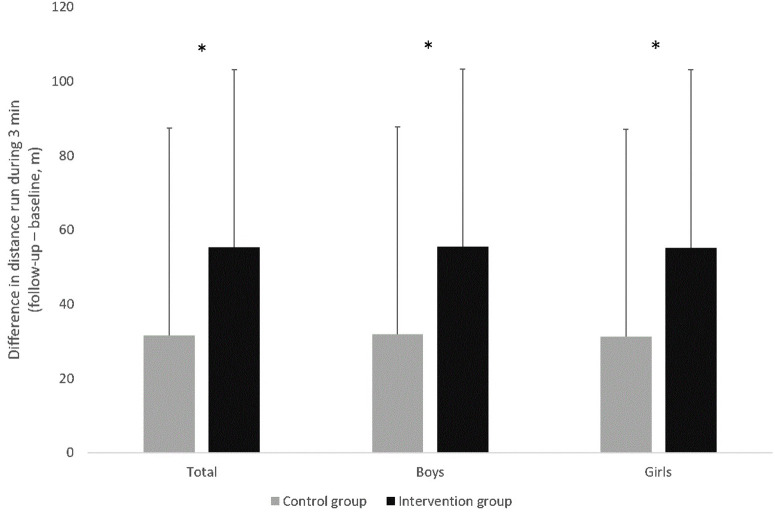
Development of endurance performance (3 min run; difference follow-up – baseline) for total sample, boys and girls as well as intervention and control group. Values are displayed as means and standard deviation, *significant difference (*p* <0.05).

As shown in Figure 1, at follow-up, the children in the intervention group ran on average 55.3 m more in 3 min than at baseline; children in the control group improved their endurance performance with 31.6 m more than the previous year significantly less, even if adjusted for gender, age, SES and baseline values $\left(F\left(1.329\right)=\;20.95,\;p<\;0.000,\;\eta_{P}^{2}=0.060\right)$. The overall model explains 37.5% of the variance $(adjusted\;R^{2}=0.375,\;F\left(6.329\right)=\;34.49,\;p<0.000,\;\eta_{P}^{2}=0.386$).

The intervention effect remains for both genders; boys $\left(F\left(1.172\right)=\;13.66,\;p\leq \;0.000,\;\eta_{P}^{2}=0.074\right)$ and girls $\left(F\left(1.152\right)=\;7.48,\;p\leq \;0.007,\eta_{P}^{2}=0.047\right)$, adjusted for age, SES and baseline values. The model for the boys explains 36.7% of the variance ($adjusted\;R^{2}=0.367,\;F\left(5.172\right)\;21.49,\;p<\text{​ }0.000,\eta_{P}^{2}=0.384$), the model for the girls explains 36.6% of the variance ($adjusted\;R^{2}=0.366,\;F\left(5.152\right)\;17.11,\;p<\;0.000,\eta_{P}^{2}=0.386$).

For the remaining three motor tests, no intervention effects were found (Table 2). Further, no gender differences in motor abilities could be seen within the study year; which applies to intervention and control group.

**Table 2:** Intervention effects for motor abilities of intervention and control group, for total sample, boys and girls [difference follow-up – baseline; values are displayed as m (sd)].

	**Intervention group**			**Control group**		
	**Boys (*n* = 149)**	**Girls (*n* = 114)**	**Total (*n* = 254)**	**Boys (*n* = 74)**	**Girls (*n* = 91)**	**Total (*n* = 165)**
3-min run [m]^a^^,^ ^b^^,^ ^c^	55.4 (48.5)	55.2 (47.2)	55.3 (47.9)	32.0 (49.0)	31.3 (55.8)	31.6 (52.7)
One-legged stand [n floor contacts]^b^	−6.4 (8.7)	−5.9 (8.1)	−6.2 (8.4)	−9.7 (8.7)	−8.8 (9.4)	−9.2 (9.1)
Sit-and-reach [cm]	−1.6 (5.5)	−0.8 (4.3)	−1.3 (5.0)	−1.1 (4.9)	−0.1 (5.6)	−0.5 (5.3)
Standing long jump [cm]	20.9 (20.0)	17.0 (20.4)	19.2 (20.2)	21.3 (21.2)	20.4 (19.8)	20.8 (20.3)

a significant difference between intervention and control group (p <0.001), adjusted for baseline values, age, gender and SES;

b significant difference for boys between intervention and control group (p <0.001), adjusted for baseline values, age and SES;

c *significant difference for girls between intervention and control group (p ≤ 0.007), adjusted for baseline values, age, and SES*.

*n, number; m, mean; sd, standard deviation, SES, socio-economic status*.

### Stratified Intervention Effects on Endurance Performance

For the intervention effect on endurance performance, it became apparent that the initial performance level $\left(F\left(1.329\right)=\;1661.01,\;p<\;0.000,\;\eta_{P}^{2}=0.329\right)$, children's gender $\left(F\left(1.329\right)=\;4.89,\;p<\;0.028,\;\eta_{P}^{2}=0.015\right)$, age $\left(F\left(1.329\right)=\;4.25,\;p<\;0.040,\;\eta_{P}^{2}=0.013\right)\;$and SES $\left(F\left(2.329\right)=\;3.82,\;p<\;0.023,\;\eta_{P}^{2}=0.023\right)$ are significantly associated with endurance performance.

#### Initial Performance

Children with low initial performance showed the greatest increase in endurance performance in control and intervention group. Yet, only children with average and high baseline performance in the 3-min run benefited from the intervention, controlled for group factor, age, gender, and SES $\left(intervention:\;{\bar{x}}_{average}=\;56.92;\;{\bar{x}}_{high}=23.76;control{:\bar{x}}_{average}=\;28.91;\;{\bar{x}}_{high}=-3.4\right)$. In both performance groups (high and average), the development of endurance performance was significantly higher than that of children in the control group with the same initial level $\left(average:F\left(1.160\right)\;=\;15.07,\;p\;{\leq \;0.000,\;\eta }_{P}^{2}\;=\;0.09;high:F\left(1.78\right)\;=\;5.54,p=\;0.02;\;\eta_{P}^{2}\;=\;0.07\right)$. In children with low motor abilities at baseline, no intervention effect was seen $\left(low:\;intervention:\;{\bar{x}}_{low}=\;87.51;control:{\bar{x}}_{low.}=\;78.10;F\;\left(1,80\right)=0.94,\;p=0.336,\;\eta_{P}^{2}=0.01\right)$.

#### Socio-Economic Status

Intervention effects are also seen in children with medium $\left(medium\;SES:\;{\bar{x}}_{IG}=61.46;\;{\bar{x}}_{CG}=25.37;\;F=\left(1.108\right)\;11.74,\;p\leq 0.001,\eta_{P}^{2}=0.10\right)$ and high SES $\left(high\;SES:\;{\bar{x}}_{IG}=56.44;\;{\bar{x}}_{CG}=39.32;\;F=\left(1.186\right)\;8.14,\;p\leq 0.005,\eta_{P}^{2}=0.04\right)$. Children with low SES in the intervention group did not significantly increase their endurance performance in comparison to those in the control group $\left(low\;SES:\;{\bar{x}}_{IG}=\;41.37;\;{\bar{x}}_{CG}=\;17.31;\;F\left(1.27\right)=\;2.26,\;p=\;0.144,\;\eta_{P}^{2}=0.08\right)$.

#### Age

Three- and four-year-old children benefited from the intervention $\left(3\;years:\;{\bar{x}}_{IG}=\;63.88;\;{\bar{x}}_{CG}=\;48.98;\;F\left(1.106\right)=\;5.60,\;p\leq \;0.02,\eta_{P}^{2}=\;0.05\right)$ ($4\;years:\;{\bar{x}}_{IG}=\;54.25;\;{\bar{x}}_{CG}=\;24.94;\;F\left(1.200\right)=\;12.01,\;p\leq \;0.001,\eta_{P}^{2}=\;0.06)$, whereas, 5-year-old children in the intervention group performed no differently than those in the control group ($5\;years:\;{\bar{x}}_{IG}=\;43.63;\;{\bar{x}}_{CG}=\;0.5;\;F\left(1.12\right)=\;3.69,\;p\leq \;0.08,\eta_{P}^{2}=0.24)$.

## Discussion

This study investigated intervention effects of the health promotion programme “Join the Healthy Boat” on motor abilities through kindergarten-based physical activity promotion. Within the 1-year study period, children in both groups (control and intervention) have improved their endurance, strength and balance; and decreased their trunk flexibility. Since early childhood is considered to be a phase of rapid motor development (48, 49), it is not surprising that the entire sample has improved in three out of four motor abilities. Physiological and physical changes support that development process (49), but during those years at kindergarten, motor abilities are practiced and completed mainly through the children's urge to play and move.

This is where the here investigated programme tries to intervene. During the intervention period, the teachers were asked to implement 20 exercise and games lessons as well as 30 ready-to-use action alternatives and ideas in order to get children to be more physically active and gain knowledge about their body and health as well as eat more healthily. In order to incorporate additional physical activity into the daily kindergarten routine, short activity games of 5–7 min each were introduced and performed twice a day. This was as much as the pedagogical advisory board, which was consulted during intervention planning, considered as possibly feasible to incorporate into a normal kindergarten routine if high and lasting implementation of the intervention was sought.

### Bounce, Flexibility, and Balance

Various kindergarten-based health promotion programmes have shown to have significant effects on bounce, balance and motor coordination in early childhood (22, 24, 26, 27). While there is a tendency for an increased development of bounce (standing long jump) in the intervention group, none of the above mentioned results can be confirmed by the here available results of “Join the Healthy Boat.”

The result of flexibility and balance (sit-and-reach and one-legged stand) indicate that children in the control group tended to perform slightly better than their counterparts in the intervention group and even showed a decrease in trunk flexibility in both groups after 1 year. The latter maybe age-related but one can only speculate to why this showed statistical significance in the intervention group. Similarly, to why – although no significant differences were found – balance tended to be slightly more pronounced in children in the control group, although they started off with worse values at baseline. Especially the short activity games of the intervention, which were implemented twice a day, have plenty of exercises to use and practice balance, as well as flexibility. According to teachers' information on implementation rates (not shown), those activities were used very regularly and children enjoyed performing those exercises.

### Endurance Performance

Further, this low-threshold intervention, mainly promoting daily physical activity in order to inter alia increase children's motor abilities found significant intervention effects for endurance performance, when controlled for gender, age, SES and baseline values; this applies to boys as well as girls, although there were more boys in the intervention group, compared to the control group. Since aerobic endurance is considered a positive predictor of well-developed health-related fitness (50), which again is positively associated with various health aspects such as cardiovascular health and weight status (51), endurance performance is looked at in most interventions assessing children's motor abilities. Like Latorre-Román et al. (25) who were able to show similar effects using a shorter (10 weeks) but much more intense programme including 3 weekly exercise lessons á 30 min focussing on the promotion of physical fitness through aerobic exercise games. Correspondingly, the health promotion programme “Ballabeina” was able to demonstrate positive intervention effects on endurance performance in kindergartens with a high proportion of children with migration background (26). This has also been confirmed for slightly older children (first and second grade of primary school) where children in the intervention group but especially boys with migration background benefited from a low-threshold school-based health and physical activity promotion and showed significantly improved endurance performance after 1 year (40, 41).

### Age Differences in Endurance Performance

Then again, looking at the significant intervention effects (for endurance performance) in more detail, it becomes apparent that children's initial motor abilities, SES, and age are significantly associated to their development of endurance performance. Three-year-olds in intervention and control group were the most likely to improve their endurance performance. Yet, significant intervention effects were found for children aged three and four, but not five. Reasons for this are unclear, especially since the materials provided for the intervention include actual activity games and exercises but also lessons for children to gain knowledge, which should theoretically address the older children more but maybe there was already a limit reached. It is difficult to valuate these findings, since only one study in kindergarten children known to date has differentiated their results according to age. In contrast to the results reported here, Birnbaum et al. (22) found intervention effects only for 4.3–5 year old children on bounce and coordination during jumping. Yet, their results and these can only be compared with each other to a limited extent, since different motor abilities were assessed.

### Differences in Endurance Performance on the Basis of Initial Performance Level

With regards to different levels of motor abilities, it was repeatedly reported that children who have particularly weak motor abilities benefit most from interventions at an early age (16, 24). Although here, the largest increase in endurance performance in both groups was seen in children with low abilities, intervention effects were only visible in children with moderate and high endurance performance at baseline. This is contraire to another German study examining effects of a physical activity promotion measure in kindergarten children, where children with low and moderate motor abilities were more likely to benefit from the intervention (24). However, the performance groups of that study (24) refer to an entire motor abilities test and have been determined on the basis of the standard values. Endurance performance levels of children in the present study were determined by quartiles of the sample distribution. In order to judge why the here assessed measure has shown effects only in children with moderate and high initial endurance performance, further qualitative details should have been collected. Whether it was that children with little interest in physical activity and possibly thus low motor ability levels were not reached because of reasons on their side, such as lack of motivation; or even on the teacher's side, e.g., excluding those children from some exercises because they know they cannot do them well so they want to spare them the disappointment. Further, maybe some of the children with low motor abilities have a pathological condition, that was not considered here.

### Differences in Endurance Performance on the Basis of Socio-Economic Background

In addition to age and initial endurance performance, children's SES was also associated to their motor abilities during the intervention period. Stratified analyses for SES showed that intervention effects on endurance performance were only significant in children from a middle and high socio-economic background; even though there was a tendency for children with low SES in the intervention group to show better results in the 3-min run. Children with low SES also showed less change in performance levels compared to those with medium and high SES; this applies to children in the intervention as well as the control group. It is already known that children with a lower SES are less physically active (4) and display worse motor abilities than children with a high SES (52, 53). Based on the present findings however, it should be considered to not only work holistically and preventive in the kindergarten setting but to try and reach those children from a socio-economically disadvantaged background as well as their parents especially with an intervention and a more specific and intense health promotion measure. “Join the Healthy Boat” already tries to reach parents with regular information about health promoting content, including letters in different languages, but possibly those letters and information do not reach parents of children from a socio-economically disadvantaged background and more joint activities, including exercises or hands-on information are needed.

While findings show that some motor abilities can be influenced by targeted promotion in early life already, and that for endurance performance “Join the Healthy Boat” can be applied in that area, there are also several limitations to be considered when interpreting the here presented results. In contrast to other physical activity promotion interventions, the duration of the individual exercise sessions (two short exercises per day and on average one game, lesson or activity per week) is below average (24–27), which may have resulted in the low intervention effects. Yet, it should be noted (as mentioned above) that this intervention was designed as a low-threshold measure so it would not interfere with the daily kindergarten routine in order to ensure high implementation rates. This however, is in conflict with a longer duration of individual physical activity sessions, as recommended by Jaščenoka and Petermann (54). On the positive side, because of the little interference of this health-promotion programme in the day-to-day routine, this intervention can be carried out from the first year at nursery, throughout kindergarten to the end of primary school, and thus support a healthy lifestyle including physical activity and well-developed motor abilities over nearly 10 years during a formative phase in children's lives. Nonetheless, this study only lasted 1year and follow-up assessments were carried out straight after a 6-week summer break with no intervention, so it can be surmised that a follow-up before the holidays (55), a shorter more intense or a multi-year low-level implementation would have had stronger effects. Further, the often discussed influence of teachers on children's development but also on the quality of health promotion measures in kindergartens (54) was not taken into consideration. On a further limiting note, only four motor tests were used in this study (speed was ignored). Motor abilities, however, are understood as the sum of all control and function processes that underlie posture and movement (56) and therefore all dimensions (endurance, strength, coordination, speed and flexibility) should be taken into account (57); again, this was owed to feasibility. Additionally, there were significantly more boys in the intervention group, compared to the control group, which might have led to a distortion of the results, at least in some aspects. Also, since “Join the Healthy Boat” encompasses physical activity promotion in order to promote motor abilities as well as nutrition advice and a reduction of screen media use in order to achieve a holistic change in health behavior, the intervention effects can probably not solely be attributed to the physical activity module of the programme, but rather that the intervention in its entirety could have led to the results. Moreover, data on physical activity, leisure time behavior and SES were taken from a parental questionnaire, which leads to the possibility of a systematic bias due to recall and social desirability bias. Finally, although the sample was large, its representativeness and transferability to other regions are limited since only children from kindergartens in south-west Germany were included and participation was voluntary. Yet, the cluster-randomized longitudinal design of the intervention study with an intervention and control group is a strength of this study.

## Conclusion

To summarize, the relationship between physical activity and motor abilities and their positive relationship to health and development of children is well-established. For kindergartens, there are only few studies examining the influence of health promotion measures on motor abilities. The present study contributes to new findings in the bespoke research gap. The kindergarten-based health promotion programme “Join the Healthy Boat” has shown positive effects on aerobic endurance, and thus on a health-related measure, in both boys and girls after a 1-year intervention. This could be achieved by a low-threshold intervention with a comparatively short duration of exercise sessions. There was no evidence of intervention effects for children with a low initial endurance performance, for children from a low socio-economical background, and for older children. In order to achieve development in terms of coordination, bounce and flexibility (which were also assessed but showed no increases), it may be necessary to have a longer lasting and/or more specific and intense intervention. This and some qualitative data on why those groups could not be reached should be investigated in future studies. The results of existing intervention studies suggest that measures with the aim to promote physical activity of kindergarten children have positive effects on endurance performance. Structured and evidence-based physical activity promotion measures should be used to support motor abilities from an early age on and across all social groups in different settings. In future interventions, motor-impaired children and children from socially disadvantaged families should be given special attention and materials should also be designed on a target-group-specific basis.

## Data Availability Statement

The raw data supporting the conclusions of this article will be made available by the authors, without undue reservation.

## Ethics Statement

The studies involving human participants were reviewed and approved by The Ministry of Education, as well as Ulm University's ethics committee (https://www.uni-ulm.de/einrichtungen/ethikkommission-der-universitaet-ulm/) have approved the study. Written informed consent to participate in this study was provided by the participants' legal guardian/next of kind; participants' verbal assent was also given. Written informed consent to participate in this study was provided by the participants' legal guardian/next of kin.

## Author Contributions

SK, OW, CL, and JS designed the study. SK, OW, and CL carried out the study. SK, LH, and BS performed the statistical analyses. SK and LH drafted the manuscript. OW, CL, BS, and JS revised the manuscript. All author contributed to the article and approved the submitted version.

## Conflict of Interest

The authors declare that the research was conducted in the absence of any commercial or financial relationships that could be construed as a potential conflict of interest.
